# Antidepressant Use is Associated with Increased Energy Intake and Similar Levels of Physical Activity

**DOI:** 10.3390/nu7115489

**Published:** 2015-11-20

**Authors:** Elsbeth Jensen-Otsu, Gregory L. Austin

**Affiliations:** 1 Division of Gastroenterology, University of Washington, 1959 NE Pacific Street, Seattle, WA 98195, USA; ejensenotsu@medicine.washington.edu; 2 Division of Gastroenterology and Hepatology, University of Colorado Anschutz Medical Campus, 12631 E. 17th Ave., Room 7619, Aurora, CO 80045, USA

**Keywords:** antidepressants, macronutrients, diet composition, food intake, physical activity

## Abstract

Antidepressants have been associated with weight gain, but the causes are unclear. The aims of this study were to assess the association of antidepressant use with energy intake, macronutrient diet composition, and physical activity. We used data on medication use, energy intake, diet composition, and physical activity for 3073 eligible adults from the 2005–2006 National Health and Nutrition Examination Survey (NHANES). Potential confounding variables, including depression symptoms, were included in the models assessing energy intake, physical activity, and sedentary behavior. Antidepressant users reported consuming an additional (mean ± S.E.) 215 ± 73 kcal/day compared to non-users (*p* = 0.01). There were no differences in percent calories from sugar, fat, or alcohol between the two groups. Antidepressant users had similar frequencies of walking or biking, engaging in muscle-strengthening activities, and engaging in moderate or vigorous physical activity. Antidepressant users were more likely to use a computer for ≥2 h/day (OR 1.77; 95% CI: 1.09–2.90), but TV watching was similar between the two groups. These results suggest increased energy intake and sedentary behavior may contribute to weight gain associated with antidepressant use. Focusing on limiting food intake and sedentary behaviors may be important in mitigating the weight gain associated with antidepressant use.

## 1. Introduction

Antidepressants are among the most widely prescribed medications, being used by more than 10% of Americans at any given time [1]. Antidepressants have long been associated with weight gain, with reported amounts ranging from 2 kg up to 14 kg [1,2,3,4,5,6,7] over follow-up periods of 6 months to 5 years. Although some classes of antidepressants cause more weight gain than others, all antidepressants have been associated with weight gain, with the notable exception of bupropion [5]. Initial observations of weight gain among individuals taking antidepressants were attributed to regaining weight that was lost as a symptom of depression. However, as more consistent data emerged, it became clear that there was additional weight gain associated specifically with the use of the antidepressant medications [2,8].

Weight gain among antidepressant users is particularly problematic as the prevalence of obesity is higher in patients with depression compared to the general population [9]. An even modest increase in weight among these individuals increases the risk of developing obesity-related conditions such as diabetes, hypertension, stroke, and obstructive sleep apnea [10,11,12]. In addition, sedentary behavior is an important variable to assess in this population given that it is independently associated with an increased risk of diabetes, cardiovascular disease, and overall mortality [13,14].

Despite the long-standing association between antidepressants and weight gain, the underlying cause of weight gain associated with antidepressant use is poorly understood. Previous studies have suggested that patients with mood disorders may have diets that are higher in fat, salt, and sugar, possibly contributing to weight gain [15,16]. However, no previous studies have investigated the effect of antidepressants alone on energy (caloric) intake, physical activity, or sedentary behaviors. Therefore, it is unclear whether the weight gain associated with antidepressants is a result of increased energy intake, decreased energy expenditure, or some combination of the two.

Given the potential negative long-term health impacts and unknown mechanism of weight gain, the aims of this study were to determine the association of antidepressant use with energy intake and physical activity using data from the National Health and Nutrition Examination Survey (NHANES). Our hypothesis was that antidepressants would be associated with increases in reported energy intake and decreased physical activity. As an exploratory objective, we also assessed macronutrient composition and hypothesized that the increase in energy intake would be the result of increases in the percentage of energy from sugar and fat.

## 2. Experimental Section

### 2.1. Population

We performed a retrospective cross-sectional analysis using data from the NHANES, a complex multistage probability sample of the United States civilian, non-institutionalized population designed to assess the health and nutritional status of adults and children in the US [17]. We used data on 4381 adults aged 20–74 years in the 2005–2006 NHANES to ascertain demographic data, medication use, diet composition, and levels of physical activity and sedentary behaviors. Interviews were used to collect demographic data. Height and weight were measured by using standardized protocols and calibrated equipment, and BMI was calculated as kg/m^2^. We excluded participants (*n* = 657) who responded “yes” to the question “Are you currently on any kind of diet, either to lose weight or for some other health-related reason?” We excluded pregnant women (*n* = 313) and underweight individuals (*n* = 91) with a BMI < 18.5 kg/m^2^. Because very few respondents described themselves as other (non-Mexican) Hispanics, other races, or of multiple races, we excluded those individuals (*n* = 247) to maximize the homogeneity of the racial groups. All participants provided informed consent, and the NHANES study protocol was approved by the National Center for Health Statistics Research Ethics Review Board. Details on the NHANES data are described in Section 2.2 through Section 2.5.

### 2.2. Medication and Tobacco Use

The Dietary Supplements and Prescription Medication Section of the Sample Person Questionnaire collects information on the use of dietary supplements, non-prescription antacid medication use, and prescription medication use [17]. Personal interviews, using the Computer-Assisted Personal Interviewing system, were conducted in the home to collect data on current medication use. All medications and dosages were recorded. There were seven sub-subcategories within the antidepressant subcategory for psychotherapeutic agents: serotonin and norepinephrine reuptake inhibitors (SNRIs), selective serotonin reuptake inhibitors (SSRIs), tricyclic antidepressants (TCAs), monoamine oxidase inhibitors (MAOIs), phenylpiperazines, tetracyclics, and miscellaneous antidepressants. Each drug was recorded and eventually entered into a 3-level nested category system (e.g., Psychotherapeutic Agents→Antidepressants→SSRI Antidepressants for citalopram). Use of antipsychotics, including typical and atypical antipsychotics, were recorded and included as a covariate. To assess tobacco use, participants were asked if they had used any tobacco or nicotine products in the previous 5 days. 

### 2.3. Energy Intake

All participants were asked to complete two 24-hour dietary recall interviews, including both weekdays and weekend days. All food items and quantities consumed by each participant from midnight to midnight on the day preceding the interviews were recorded. The first interview was conducted in person. The second interview was collected by telephone 3–10 days later, although not on the same day of the week as the in-person interview. The dietary recalls used the Automated Multiple Pass Method, which is designed to increase the efficiency and accuracy of the 24-hour recall by including a thorough compilation of standardized food-specific questions and possible responses [18]. Participants were given a set of measuring guides to help in reporting food amounts during both interviews, as well as a food model booklet to assist in reporting food amounts during the telephone interview. The data were used to calculate mean total energy intake (kcal/day) and the proportion of calories from macronutrients including sugar, non-sugar carbohydrates, saturated fatty acids (SFAs), polyunsaturated fatty acids (PUFAs), monounsaturated fatty acids (MUFAs), protein, and alcohol with the use of the USDA’s Food and Nutrient Database for Dietary Studies [18]. We excluded individuals (*n* = 34) who had implausible energy intake for those with energy intake <500 kcal/day or >5000 kcal/day [19].

### 2.4. Physical Activity and Sedentary Behaviors

All participants were asked a series of questions about their physical activity. This included the question, “Compared with most men/women your age, would you say that you are more active, less active, or about the same?” Participants were also asked if they participated in specific physical activities (not in the workplace) in the previous 30 days including walking, bicycling and muscle strengthening activities. If they answered yes, they were asked about the frequency and the average duration of time they engaged in those activities. Responses for walking and bicycling were recorded as daily, weekly, or monthly and were subsequently converted into the total amount of time respondents engaged in these activities in the past 30 days. The outcome for the frequency of walking or biking per 30 days was dichotomized into those who engaged in these activities for at least 150 min in the last 30 days. The outcome for whether participants engaged in any moderate or vigorous physical activity for at least 10 min within the past 30 days was recorded in NHANES as a dichotomous response. Similarly, the outcome of whether participants engaged in any muscle strengthening activity in the past 30 days was recorded in NHANES as a dichotomous response. Sedentary behaviors were assessed by the questions, “Over the past 30 days, on average about how many hours per day did you sit and watch TV or videos?” and “Over the past 30 days, on average how many hours per day did you use a computer or play computer games?” The responses for watching TV or videos were dichotomized into those who watched TV for 3 or more hours per day and those who watched less than 3 h per day. The responses for computer use were dichotomized into those who used a computer for 2 or more hours per day and those who used a computer for less than 2 h per day.

### 2.5. Depression Symptoms

All participants were asked to complete the Brief Patient Health Questionnaire-9 (PHQ-9), which is a nine question survey based on the DSM-IV (Diagnostic and Statistical Manual of Mental Disorders) criteria for major depressive disorder. The PHQ-9 assesses specific symptoms of depression, including mood, sleep, energy, appetite, concentration, suicidal ideation, and symptoms of depression and is scored on a 0–27 point scale. This survey is validated as a screening tool for major depression and other depressive disorders in primary care settings as well the general population [20,21].

### 2.6. Covariates

All analyses were adjusted for age, gender, education, race, BMI category (normal-weight, overweight, or obese), PHQ-9 score, use of tobacco/nicotine in previous 5 days, use of insulin, use of non-insulin diabetic medications, use of lipid-lowering medications, use of antipsychotics, and total number of non-psychiatric/non-diabetic medications. The response to the question “Compared with most men/women your age, would you say that you are more active, less active, or about the same?” was also included as a covariate for the energy intake outcome because of its strong association with BMI, several other covariates, and energy intake. BMI categories were defined as normal weight (18.5 < BMI < 25), overweight (25 ≤ BMI < 30), and obese (BMI ≥ 30). Education was dichotomized into those who had attended at least some college and those who had a high school degree or less. We coded race as non-Hispanic white, non-Hispanic black, and Mexican-American. Depression symptoms were included as a covariate using the PHQ-9 score (0–27) as a continuous variable. Antipsychotics were also included as a covariate due to their association with both weight gain and antidepressant use. Because there were very few patients (*n* = 4) taking typical antipsychotics, we included both typical and atypical antipsychotics as a single covariate.

### 2.7. Data Analysis

Statistical analyses were performed using Stata software (version 10.1; StataCorp, College Station, TX, USA). We used survey commands and applied the appropriate sample weights for the data to account for the unequal probabilities of selection. Means (±S.E.) for percentages and total calories from sugar, non-sugar carbohydrates, protein, SFAs, MUFAs, PUFAs, and alcohol were calculated separately for individuals who reported taking antidepressant medications and those who were not taking these medications. Univariate comparisons by antidepressant use were performed with linear or logistic regression. We used multivariable regression analyses (adjusted for the above covariates) to assess the relationship between antidepressant use and the primary outcomes: daily energy intake, self-assessment of physical activity compared to age/gender-matched peers (more, less, or the same), walking or biking at least 150 minutes (in the past 30 days), engaging in at least 10 consecutive minutes of any moderate/vigorous physical activity (in the past 30 days), engaging in any muscle strengthening activities (in the past 30 days), and sedentary behaviors. Because bupropion has been associated with weight loss, we analyzed the association of antidepressant use with the outcomes both with and without those taking bupropion as well as the bupropion group separately. Because there was no effect modification by bupropion on the association of antidepressant use with any of the outcomes, we report the analyses in which patients taking bupropion are combined with users of all other antidepressants.

## 3. Results

### 3.1. Demographics

Univariate analyses of demographic characteristics of those participants taking and not taking antidepressants are shown in Table 1. Of the 221 individuals who reporting taking antidepressant medications, 148 were taking SSRIs, 38 were taking SNRIs, eight were taking TCAs, and 42 were taking other (non-SSRI/SNRI/TCA) antidepressants (the majority of which was bupropion (*n* = 29)). There were 15 individuals who reported taking more than one antidepressant, with eight individuals reporting use of an SNRI/SSRI with a TCA and 7 individuals reporting use of an SNRI/SSRI with other antidepressants. Those on antidepressants were more likely to be older, female, and white. Patients in the antidepressant group had a higher average BMI and were more likely to be obese (both *p* ≤ 0.006). Antidepressant users had a higher PHQ-9 score (*p* < 0.001) and were more likely to be taking antipsychotics (*p* = 0.048). In addition, antidepressant users took on average two additional non-psychiatric medications compared to those not on antidepressants (*p* < 0.001). There was no difference in education level, family income, or current tobacco/nicotine use between the two groups.

**Table 1:** Patient Demographics by Antidepressant use from the 2005–2006 National Health and Nutrition Examination Survey (NHANES).

	Antidepressant Non-Users (*n* = 2818)	Antidepressant Users (*n* = 221)	*p*-Value ^1^
Age (mean ± S.E.), years	43.5 ± 0.6	48.9 ± 1.0	<0.001
Gender (% Women)	46.6	72.6	<0.001
Race (% White non-Hispanic)	70	89.1	<0.001
Completed some college (%)	58.5	55.3	0.412
Annual Family Income < $20,000 (%)	15.1	16.2	0.589
Annual Family Income $20,000 to $75,000 (%)	54.9	52.1	0.564
Annual Family Income ≥ $75,000 (%)	30.1	31.7	0.724
BMI (mean ± S.E.), kg/m^2^	28.0 ± 0.3	29.8 ± 0.5	0.004
Normal-Weight (%)	32.3	30.3	0.693
Overweight (%)	33.9	24.7	0.040
Obese (%)	31.2	42.2	0.006
Mean PHQ-9 Depression Score (Scale 0–27)	2.3 ± 0.1	4.7 ± 0.4	<0.001
Used Tobacco/Nicotine in the last 5 days (%)	31.2	33.5	0.422
Antipsychotic use (%)	0.6	5.9	0.048
Non-psychiatric medications (mean ± S.E.), n	1.2 ± 0.1	3.2 ± 0.2	<0.001

^1^ *p*-values are for the overall F-test for each variable in univariate analysis.

### 3.2. Energy Intake and Diet Composition

Overall, patients taking antidepressants had similar diet composition compared to those not taking antidepressants (Table 2). There was no difference in percent calories from sugar, fat, or alcohol between the two groups. Individuals taking antidepressants did consume a reduced proportion of calories from protein, but the absolute difference was small (14.9% of total caloric intake for antidepressant users *vs.* 15.5% for non-users).

**Table 2:** Diet Composition by Antidepressant use from the 2005–2006 NHANES. ^1^

Macronutrient ^3^ (% Energy)	Antidepressant Non-Users (*n* = 2818)	Antidepressant Users (*n* = 221)	*p*-Value ^2^
Carbohydrate (%)	48.1 ± 0.2	48.7 ± 0.6	0.484
Sugar (%)	21.8 ± 0.3	23.0 ± 0.7	0.118
Non-sugar carbohydrates (%)	26.2 ± 0.2	25.7 ± 0.5	0.413
Protein (%)	15.5 ± 0.1	14.9 ± 0.3	0.033
Total Fat (%)	33.2 ± 0.2	33.8 ± 0.5	0.434
SFAs (%)	11.1 ± 0.1	11.6 ± 0.2	0.191
MUFAs (%)	12.2 ± 0.1	12.3 ± 0.2	0.650
PUFAs (%)	7.1 ± 0.1	7.1 ± 0.2	0.785
Alcohol (%)	3.2 ± 0.2	2.6 ± 0.4	0.219

^1^ Values are means ± S.E. of percentages; ^2^ *p*-values are for the overall F-test for each variable in univariate analysis; ^3^ SFA: saturated fatty acids; MUFA: monounsaturated fatty acids; PUFA: polyunsaturated fatty acids.

**Figure 1:**
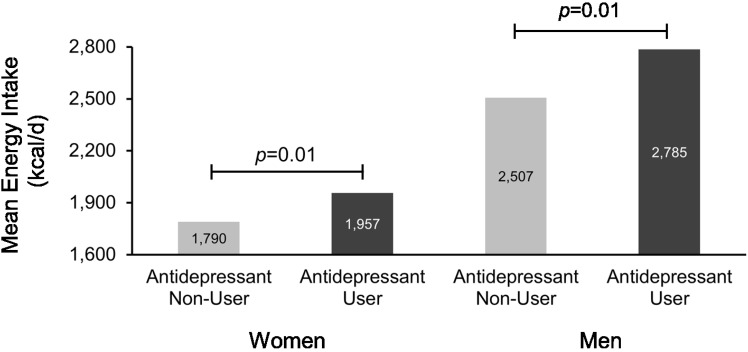
Antidepressant use is associated with increased energy intake (kcal/day) among adult men and women in the 2005–2006 National Health and Nutrition Examination Survey. Adjusted for Patient Health Questionnaire-9 depression score, tobacco/nicotine use, antipsychotic use, use of insulin, use of non-insulin diabetic medications, use of lipid-lowering medications, number of non-psychiatric/non-diabetic medications, self-assessed physical activity level, BMI category, education, race/ethnicity, and age.

After adjusting for all covariates, antidepressant users reported consuming an additional (mean ± S.E.) 215 ± 73 kcal/day compared to non-users (*p* = 0.01). Similar increases in daily energy intake among antidepressant users were seen in stratified analyses of men and women (Figure 1). Patients taking bupropion had a similar increase in energy intake compared to patients taking other antidepressants and the association of antidepressant use with energy intake was essentially identical when patients taking bupropion were excluded from analysis.

There was no effect of depression symptoms (as measured by the PHQ-9) on energy intake. A 1-point increase in the PHQ-9 score (which ranged from 0 to 27) was associated with a decrease in daily energy intake of −0.8 kcal (*p* = 0.85). A 1-point increased in PHQ-9 was not significant in men (−4.6 kcal, *p* = 0.452) or women (−0.6 kcal, *p* = 0.92). PHQ-9 scores were not associated with changes in the percentage of calories from carbohydrates (both sugars and non-sugar carbohydrates), fats (SFAs, MUFAs, and PUFAs), protein, or alcohol.

### 3.3. Physical Activity and Sedentary Behavior

**Figure 2:**
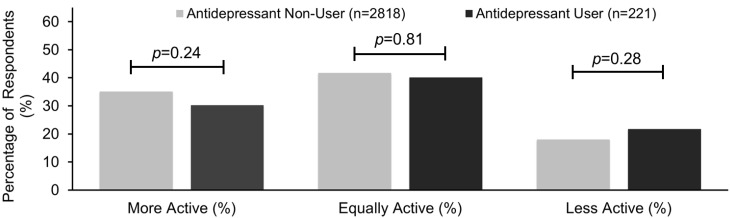
Adults taking antidepressants self-report that they were as physically active compared to their age and gender-specific peers from the 2005–2006 National Health and Nutrition Examination Survey. Adjusted for Patient Health Questionnaire-9 depression score, tobacco/nicotine use, antipsychotic use, use of insulin, use of non-insulin diabetic medications, use of lipid-lowering medications, number of non-psychiatric/non-diabetic medications, self-assessed physical activity level, BMI category, education, race/ethnicity, and age.

After adjusting for depressive symptoms, antidepressant users had similar levels of self-reported physical activity and sedentary behavior. In response to the question, “Compared with most men/women your age, would you say that you are more active, less active, or about the same?”, antidepressant users had similar responses to those not taking antidepressants (Figure 2). Interestingly, each 1-point increase in the PHQ-9 score was associated with an increase in the likelihood that the individuals would report that they are less active compared to their age/gender-matched peers (OR 1.13; 95% CI: 1.08–1.18, *p* < 0.001).

**Figure 3:**
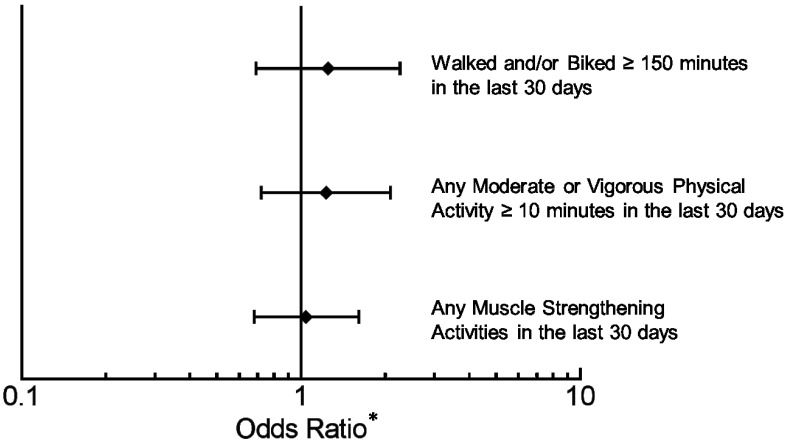
Adults taking antidepressants had similar self-reported measures of physical activity in the 2005-2006 National Health and Nutrition Examination Survey compared to those not taking antidepressants. Adjusted for Patient Health Questionnaire-9 depression score, tobacco/nicotine use, antipsychotic use, use of insulin, use of non-insulin diabetic medications, use of lipid-lowering medications, number of non-psychiatric/non-diabetic medications, self-assessed physical activity level, BMI category, education, race/ethnicity, and age. *Odds Ratio (OR) with 95% CI’s of antidepressant users compared to non-users.

When individuals were asked about engagement in particular activities in the preceding 30 days, antidepressant users reported similar levels of walking, biking, muscle strengthening, and any moderate or vigorous activity lasting at least consecutive 10 min (Figure 3). The overall percentage of individuals who walked or biked at least 150 min in the past 30 days was 18.0% (95% CI: 15.6%–20.4%), with antidepressant users reporting a similar frequency (Figure 3) compared to those not taking an antidepressant (OR 1.23 95% CI: 0.68–2.23). The overall percentage of individuals who participated in at last ten consecutive minutes of moderate or vigorous physical activity in the prior 30 days was 68.5% (95% CI: 64.8%–72.2%), and antidepressant users reported a similar frequency compared to non-users (OR 1.22; 95% CI: 0.72–2.07). The overall percentage of individuals who participated in muscle-strengthening activities within the previous 30 days was 30.6% (95% CI: 26.4%–34.7%), with antidepressant users as likely to have engaged in these activities (OR 1.05; 95% CI: 0.68–1.63). The PHQ-9 score was not associated with having walked or biked for greater than 150 min (*p* = 0.464) or having engaged in muscle-strengthening activities (*p* = 0.498) in the past 30 days. However, each 1-point increase in the PHQ-9 score was associated with a decrease in the odds of having engaged in at least 10 consecutive minutes of moderate or vigorous physical activity (OR 0.94, 95% CI: 0.91–0.97; *p* < 0.001).

Regarding sedentary behavior, the overall percentage of individuals who reported spending two or more hours per day using a computer or playing computer games was 16.3% (95% CI: 14.1%–18.4%), with antidepressant users reporting a higher frequency compared to non-users (OR 1.77; 95% CI: 1.09–2.90). The PHQ-9 score was not associated with time spent using a computer or playing computer games (*p* = 0.16). The overall percentage of individuals who reported spending three or more hours per day watching TV or videos was 36.6% (95% CI: 33.9%–39.3%), with antidepressant users reporting a similar frequency compared to non-users (OR 0.80; 95% CI: 0.56–1.14). Each 1-point increase in the PHQ-9 score was associated with an increase in the odds of spending 3 or more hours a day watching TV or videos (OR 1.04; 95% CI: 1.01–1.07).

## 4. Discussion

Antidepressants are one of the most commonly prescribed classes of medications in the United States, and they are associated with weight gain in a population that has a higher baseline prevalence of obesity [1,5,10]. While the association of antidepressants with increased body weight is well known, the factors leading to weight gain have been poorly described. The purpose of this study was to identify the cause of weight gain associated with antidepressants using data from the NHANES. After adjusting for confounding variables (including being overweight or obese, antipsychotic use, and depression symptoms), antidepressant users reported significantly higher daily energy intake of more than 200 kcal per day and some increase in sedentary behavior. Antidepressant users had similar levels of specific physical activities compared to non-users. Our results suggest that weight gain associated with antidepressants may be caused by both increased energy intake as well as increased sedentary behavior.

The macronutrient composition of the diet was quite similar between antidepressant users and non-users. No prior data exists on the effect of antidepressants on diet composition. Individuals on antidepressants did have a small decrease in percent calories from protein (14.9% *vs.* 15.5%). While prior data has shown that a decreased percentage of energy intake from protein is associated with higher total energy intake, the small decrease observed in antidepressant users in this study would not account for the additional 215 kcal/day [22].

No prior studies have reported on differences in energy intake between antidepressant users and non-users. Importantly, the association of antidepressants with increased energy intake in this study was observed even after controlling for antipsychotics, which are strongly associated with weight gain [16]. Obesity and depression symptoms are also associated with increased weight and were more prevalent among antidepressant users [10,23]. However, the increased energy intake among antidepressant users was independent of both depression symptoms as well as being overweight or obese. The level of additional caloric intake associated with antidepressant use in this study could quite feasibly cause weight gain in line with the 2 kg to 14 kg that previous studies have associated with antidepressants, especially considering the average use of an antidepressant is months to years [1,2,3,4,5,6,7].

Antidepressant users reported similar levels of physical activity, but increased amounts of some sedentary behaviors. Although many reports have evaluated the relationship of depression with physical activity and sedentary behaviors, no prior studies have described the association of antidepressants alone on activity levels [24,25,26]. While antidepressant users had similar levels of specific measures of physical activity including walking, biking, and muscle strengthening activities, they were also more likely to use computers for 2 or more hours per day. This was independent of depression symptoms, which were associated with some increase in sedentary behaviors [27]. Not only does increased sedentary behavior likely contribute to weight gain associated with antidepressants, but the observation that antidepressants are independently associated with increased sedentary behavior has other potential health impacts as sedentary behaviors are associated with an increased risk of cardiovascular disease, diabetes, and overall mortality [13,14].

Although this study has several strengths, there are some limitations. One limitation is the use of patient recall for diet and physical activity measurements. Underreporting of caloric intake is a common problem with dietary recall measures and it is possible that one group may have underreported more than others [28]. However, we controlled for current depression symptoms and being overweight or obese in all of our outcome models. Future studies could use more objective measures of both energy intake and physical activity, for example using wearable devices to measure activity and sedentary behaviors. Another limitation is the observational nature of the study may be associated with there being unmeasured cofounders that may explain the associations identified in this study. However, known cofounders associated with weight gain and antidepressants including antipsychotics, tobacco use, depression symptoms, and obesity were included in our analysis. An additional limitation is that we were unable to evaluate the underlying mechanisms for how antidepressants increase energy intake. Possible areas for future research could include whether antidepressants increase appetite, decrease post-prandial satiety, or mediate increase energy intake through other mechanisms.

## 5. Conclusions

Given the potentially adverse effects of weight gain in a population with already higher than average rates of obesity, identifying the cause of weight gain associated with antidepressants has important clinical applicability. The results of our study demonstrate that individuals taking antidepressants have similar overall levels of physical activity and diet composition, but they have increased total energy intake and possibly increased sedentary behavior. Closer weight monitoring and more intense lifestyle counseling, with an emphasis on monitoring and restricting both caloric intake and sedentary behaviors, may be important in mitigating weight gain associated with antidepressants.
